# Genomic epidemiology and evolutionary dynamics of the Omicron variant of SARS-CoV-2 during the fifth wave of COVID-19 in Pakistan

**DOI:** 10.3389/fcimb.2024.1484637

**Published:** 2024-10-22

**Authors:** Aroona Razzaq, Cyrollah Disoma, Sonia Iqbal, Ayesha Nisar, Muddassar Hameed, Abdul Qadeer, Muhammad Waqar, Sardar Azhar Mehmood, Lidong Gao, Sawar Khan, Zanxian Xia

**Affiliations:** ^1^ Department of Cell Biology, School of Life Sciences, Central South University, Changsha, China; ^2^ Institute of Molecular Biology and Biotechnology, The University of Lahore, Lahore, Pakistan; ^3^ Key Laboratory of Genetic Evolution & Animal Models, Kunming Institute of Zoology, Chinese Academy of Sciences, Kunming, Yunnan, China; ^4^ Center for Zoonotic and Arthropod-borne Pathogens, Virginia Polytechnic Institute and State University, Blacksburg, VA, United States; ^5^ Department of Zoology, Hazara University, Mansehra, Pakistan; ^6^ Hunan Workstation for Emerging Infectious Disease Control and Prevention, Chinese Academy of Medical Sciences, Hunan Provincial Center for Disease Control and Prevention, Changsha, China

**Keywords:** COVID-19, Omicron, genomic epidemiology, SARS-CoV-2, VOCs

## Abstract

**Introduction:**

The coronavirus disease 2019 (COVID-19) pandemic, caused by severe acute respiratory syndrome coronavirus 2 (SARS-CoV-2), has posed extraordinary challenges to global health systems and economies. The virus’s rapid evolution has resulted in several variants of concern (VOCs), including the highly transmissible Omicron variant, characterized by extensive mutations. In this study, we investigated the genetic diversity, population differentiation, and evolutionary dynamics of the Omicron VOC during the fifth wave of COVID-19 in Pakistan.

**Methods:**

A total of 954 Omicron genomes sequenced during the fifth wave of COVID-19 in Pakistan were analyzed. A Bayesian framework was employed for phylogenetic reconstructions, molecular dating, and population dynamics analysis.

**Results:**

Using a population genomics approach, we analyzed Pakistani Omicron samples, revealing low within-population genetic diversity and significant structural variation in the spike (S) protein. Phylogenetic analysis showed that the Omicron variant in Pakistan originated from two distinct lineages, BA.1 and BA.2, which were introduced from South Africa, Thailand, Spain, and Belgium. Omicron-specific mutations, including those in the receptor-binding domain, were identified. The estimated molecular evolutionary rate was 2.562E-3 mutations per site per year (95% HPD interval: 8.8067E-4 to 4.1462E-3). Bayesian skyline plot analysis indicated a significant population expansion at the end of 2021, coinciding with the global Omicron outbreak. Comparative analysis with other VOCs showed Omicron as a highly divergent, monophyletic group, suggesting a unique evolutionary pathway.

**Conclusions:**

This study provides a comprehensive overview of Omicron’s genetic diversity, genomic epidemiology, and evolutionary dynamics in Pakistan, emphasizing the need for global collaboration in monitoring variants and enhancing pandemic preparedness.

## 1 Introduction

The coronavirus disease 2019 (COVID-19) pandemic, caused by the severe acute respiratory syndrome coronavirus 2 (SARS-CoV-2), has presented unprecedented challenges to global health systems and economies (Filip et al., 2022; Naseer et al., 2022; Nayak et al., 2022; Albreht, 2023; Katole, 2023; McKibbin and Fernando, 2023). Since its emergence in late 2019 (Wu et al., 2020; Zhu et al., 2020), the virus has undergone rapid evolution, leading to the appearance of several variants of concern (VOCs) characterized by increased transmissibility, altered pathogenicity, and potential immune escape (Otto et al., 2021; Scovino et al., 2022; Carabelli et al., 2023; Markov et al., 2023; Zhao et al., 2024). Genomic mutations have led to the emergence of such variants, which significantly impacted the pandemic by increasing morbidity and mortality rates worldwide (Khandia et al., 2022; Markov et al., 2023). Among these, the Alpha, Delta, and Omicron VOCs have been particularly noteworthy due to their unique genetic profiles and effects on disease transmission and severity. The Alpha VOC, also designated B.1.1.7, was initially detected in the United Kingdom and rapidly propagated globally (Bayarri-Olmos et al., 2021; Stadtmüller et al., 2022). This variant is marked by mutations in the spike protein, especially N501Y, which contributed to its heightened transmissibility and raised concerns about its ability to circumvent immunity acquired from previous infection or vaccination (Bayarri-Olmos et al., 2021). Subsequently, the Delta VOC (B.1.617.2) became predominant, driving increases in COVID-19 cases worldwide. Key mutations in Delta, such as L452R and P681R, were linked to its enhanced infectivity and potentially greater severity compared to earlier strains (Zhan et al., 2022).

Among the VOCs, the Omicron variant (B.1.1.529), first identified in South Africa in late 2021 (Karim and Karim, 2021; Tegally et al., 2022; Viana et al., 2022), has swiftly become a dominant strain worldwide due to its extensive spike protein mutations. With over 50 sub-lineages identified, Omicron has shown rapid evolution and diversification since its initial discovery, posing significant challenges for vaccine efficacy and diagnostics (Alkhatib et al., 2022; Chen et al., 2022; VanBlargan et al., 2022; Zhou et al., 2022; Piubelli et al., 2024). This variant is particularly notable for its high number of spike protein mutations (Callaway, 2021; Liu et al., 2024), which is the primary target for neutralizing antibodies elicited by both natural infection and vaccination. These mutations have raised concerns about reduced vaccine efficacy and increased potential for breakthrough infection and reinfection. Initial studies suggest that Omicron exhibits enhanced transmissibility compared to previous VOCs, likely due to its ability to evade immune responses (Jung et al., 2022; Willett et al., 2022; Reuschl et al., 2024). The rapid global spread of Omicron underscores the necessity for a thorough understanding of its genomic characteristics and evolutionary dynamics across the globe, which is critical for monitoring the virus’s evolution and implementing effective public health strategies to mitigate its spread.

Genomic surveillance has been pivotal in monitoring the evolution of SARS-CoV-2 variants (Robishaw et al., 2021; Tosta et al., 2023). Through the analysis of viral genomes from diverse geographical locations and temporal points, researchers have pinpointed critical mutations linked to alterations in viral phenotype and transmission dynamics. For example, mutations in the spike protein, such as D614G, have been associated with increased viral infectivity and transmissibility (Korber et al., 2020). Furthermore, the detection of variants with mutations in the receptor-binding domain (RBD), such as E484K found in the Beta variant, has heightened concerns about immune evasion and vaccine resistance (Wang et al., 2021). Epidemiological studies leveraging genomic data have clarified the evolutionary relationships of the virus by employing whole-genome sequencing (WGS) and phylogenetic analysis. These methods have provided crucial insights into the origins and spread of the COVID-19 pandemic across different countries (Chrysostomou et al., 2023; El Mazouri et al., 2024; Gerashchenko et al., 2024; Linosefa et al., 2024). Understanding the genomic epidemiology, evolutionary history, and population dynamics of variants of concern (VOCs), such as Omicron, is critical for effective public health strategies and future pandemic preparedness. Genomic surveillance, particularly through whole-genome sequencing (WGS) and population genomics analyses, has emerged as a powerful tool in this regard. These methods offer profound insights into the transmission patterns, genetic diversity, and evolutionary history of viral pathogens. Such comprehensive analyses are indispensable for informing targeted interventions and controlling the spread of the virus.

Pakistan, like many other countries, has experienced multiple waves of COVID-19 (Umair et al., 2022a, Umair et al., 2022b; Ahmad et al., 2023; Arif and Mahmood, 2023; Javed et al., 2024), with the Omicron variant contributing significantly to the surges in cases (Khan et al., 2022; Bukhari et al., 2023). Despite the substantial impact of Omicron on the Pakistani population, detailed studies focusing on its molecular epidemiology and evolutionary trajectory within this region are limited. This gap in knowledge necessitates a comprehensive analysis to provide deeper insights into the genetic diversity, population structure, and evolutionary dynamics of the Omicron variant in Pakistan. By leveraging WGS data, we implemented a population genomics approach to elucidate the evolutionary history, population dynamics, spread patterns, and genomic epidemiology of Omicron VOC within the Pakistani context. Our analysis reveals patterns of genetic diversity and population dynamics of Omicron, highlighting notable similarities and differences compared to other VOCs, particularly the Delta variant and the earlier Wuhan strain. By providing insights into the origins, spread, and mutation patterns of the Omicron variant, this study enhances our understanding of the evolutionary dynamics of the Omicron wave in Pakistan.

## 2 Materials and methods

### 2.1 Data set

The fifth wave of COVID-19 in Pakistan occurred from December 2021 to early April 2022 and was caused by the Omicron VOC. Nationwide data on the Omicron VOC were collected during this period. A total of 954 whole-genome sequences and associated metadata (**Supplementary Table 1**) for the Omicron VOC were obtained from the GISAID database (https://www.gisaid.org/) by applying filters for “Omicron VOC,” “Pakistan,” and the relevant date range. After rigorous quality assurance (by sequence alignment and visually inspecting), sequences that were incomplete or ambiguous were excluded, resulting in 877 complete sequences being selected for subsequent analyses. For phylogenetic reconstructions and comparative analysis, nucleotide sequencing data were sourced from the GenBank and GISAID databases. This dataset included Omicron sequences from various countries, the ancestral Wuhan strain, other VOCs, as well as sequences from SARS-CoV-1 and coronaviruses found in bats and pangolins. Additionally, nucleotide sequences from the ancestral Wuhan strain, Omicron from various countries, other VOCs, SARS-CoV-1, and coronaviruses from bats and pangolins (**Supplementary Table 2**) were retrieved from the GenBank database (https://www.ncbi.nlm.nih.gov/genbank/).

### 2.2 Sequence alignment and mutation analysis

The software tools SnapGene (https://www.snapgene.com) and BioEdit (Hall, 1999) were used to handle the sequencing data. The Wuhan-1 sequence of SARS-CoV-2 (GenBank accession number: NC_045512) served as the reference genome, and multiple sequence alignment (MSA) was performed against it using MUSCLE algorithm in software Unipro UGENE v50.0 (https://ugene.net/). For Spike protein (S) based analyses, the sequences were trimmed according to the genomic coordinates (nucleotide positions 21563 to 25384) corresponding to the Spike protein on the reference genome. Single nucleotide polymorphisms (SNPs) and insertions-deletions (InDels) were visualized using BioEdit software.

### 2.3 Genetic diversity and haplotype network analysis

The MSA files were analyzed to compute population genetic parameters including the number of haplotypes (H), nucleotide diversity (Pi), the average number of nucleotide differences (k), genetic differentiation (FST), Tajima’s D, haplotype diversity (Hd), and Watterson’s estimator (θw) using DnaSP6 software (Rozas et al., 2017). The FST score matrix was used to visualize the heatmap of population structure in the ClustVis tool (https://biit.cs.ut.ee/clustvis/) by clustering both rows and columns using correlation distance and average linkage models. A median-joining haplotype network was constructed using PopART software (Leigh and Bryant, 2015). The MSA file (in Nexus format) of SARS-CoV-2 sequences was used as input.

### 2.4 Phylogenetic reconstructions and molecular dating

The ModelFinder tool (Kalyaanamoorthy et al., 2017) was used to determine the best-fit model of evolution for phylogenetic reconstruction. The results from the Akaike and Bayesian Information Criteria (AIC and BIC) were employed to select the best-fit evolutionary models for the dataset.

Before executing the Bayesian framework, the dataset was refined to reduce its size, thereby saving the extensive computational time and resources required by Bayesian simulations. To achieve an optimal tree topology, sequences were initially analyzed using maximum-likelihood-based phylogenetic reconstructions with the IQ-TREE2 tool (Minh et al., 2020).

Time-calibrated phylogenies were reconstructed using tip dating within a Bayesian framework using BEAST2 (Bouckaert et al., 2014) and Markov chain Monte Carlo (MCMC) algorithms (Drummond et al., 2002, Drummond et al., 2003, Drummond et al., 2005, Drummond et al., 2006, Drummond et al., 2012). A general time-reversible (GTR) site model with a fixed rate and a strict molecular clock of 8.4×10^-4^ mutations/site/year (https://nextstrain.org/) was applied. The MCMC chains was executed for 1 billion steps with parameter sampling every 1,000 steps. Trace files generated by MCMC were evaluated using the Tracer package (Rambaut et al., 2018), ensuring effective sample sizes (ESS) of 200 or greater. The maximum clade credibility chronogram was extracted using TreeAnnotator and visualized in Figtree (http://tree.bio.ed.ac.uk/software/figtree/).

### 2.5 Population dynamics

To reflect population dynamics over time, a Bayesian skyline plot (BSP) (Drummond et al., 2005) was employed using BEAST2 with MCMC algorithms. The BSP was generated for both Omicron-only and all VOCs of SARS-CoV-2, using a strict molecular clock with a fixed mutation rate of 8.4×10^-4^ mutations/site/year. The MCMC chains was executed for 5 billion iterations, with parameter sampling occurring every 1,000 steps. The resulting log file was analyzed in Tracer for quality assurance and BSP extraction, ensuring ESS values of 200 or greater. The BSP was then visualized and extracted using Tracer (Rambaut et al., 2018).

### 2.6 Protein 3D structure analysis

The impact of mutations was visualized by reconstructing the 3D structures of proteins using the SWISS-MODEL server (https://swissmodel.expasy.org/). Structural figures were prepared by loading the resulting models into the AutoDock molecular graphics system (https://autodock.scripps.edu/), and PyMOL software (https://www.pymol.org/) focusing on mutations in the receptor binding domain (RBD) of the Spike protein.

## 3 Results

### 3.1 The fifth wave of COVID-19 in Pakistan and Omicron data

**Figure 1:**
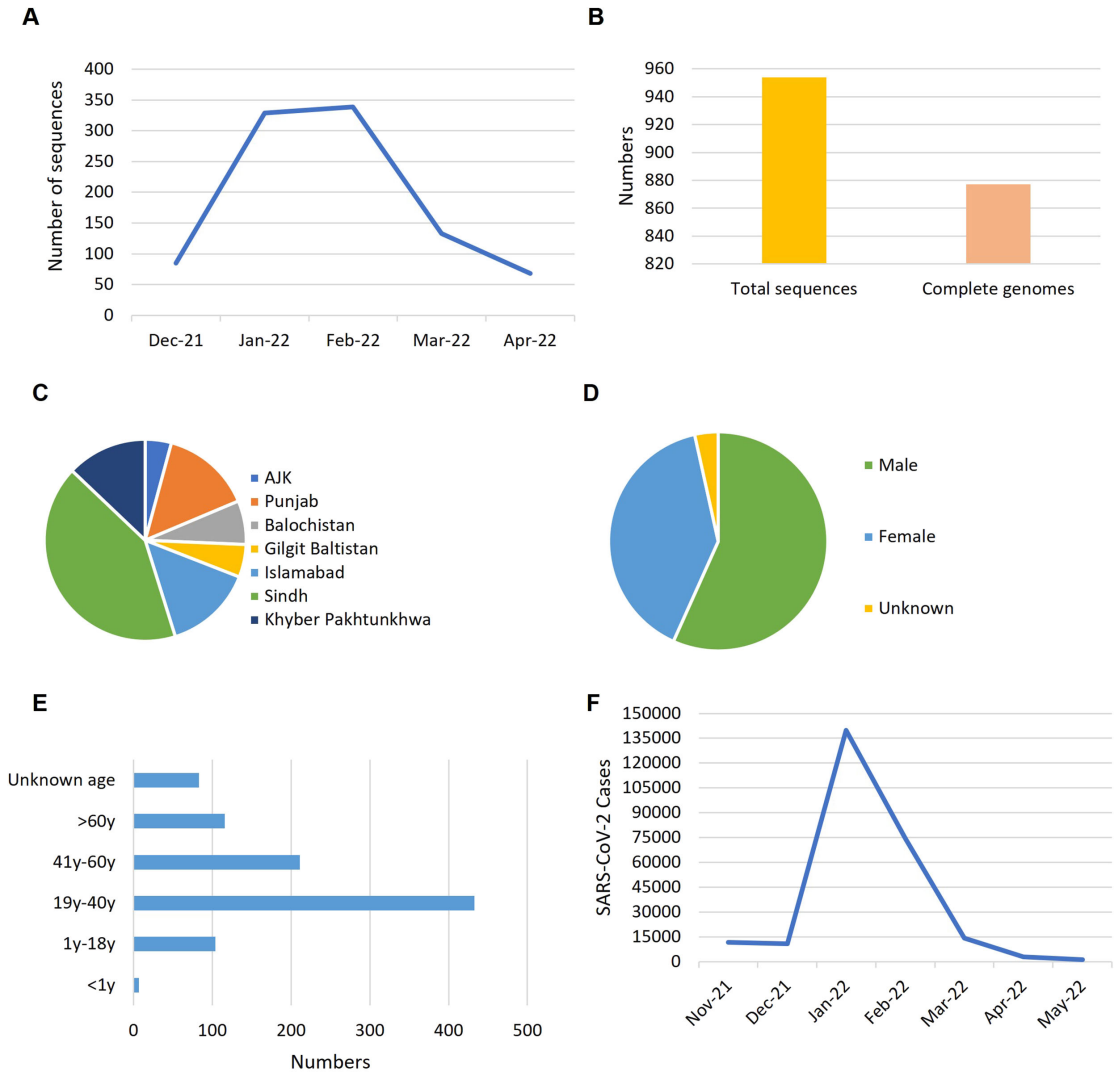
The trends reflected by Omicron data during the fifth wave of COVID-19 in Pakistan. **(A)** Rate of data reporting. **(B)** Numbers of genomic sequences. **(C)** Distribution of samples across the country. **(D)** Gender ratio of samples. **(E)** Age distribution of the samples. **(F)** Epidemiological data of COVID-19 from October 2021 to May 2022 (source: https://coronavirus.jhu.edu/region/pakistan). Time line: Nove-21, November 2021; Dec-21, December 2021; Jan-22, January 2022 and so on.

To understand the trends of Omicron data during the fifth wave of COVID-19 in Pakistan, we analyzed the genomic data based on the collection date and sample location from December 2021 to April 2022 across the country (**Figure 1**). The rate of data reporting during this period reflected a typical epidemic pattern, with cases rising quickly, plateauing, and then declining (**Figure 1A**). Heterogeneity and variability were observed in the frequency and distribution of data across the country in terms of location, gender, and age groups. Out of 954 total sequences, 877 represented complete genome coverage (**Figure 1B**). The majority of samples were from Sindh province (42%), followed by Punjab (15%), Islamabad (14%), and Khyber Pakhtunkhwa (13%) (**Figure 1C**). Gender distribution of the samples showed 57% male and 40% female, with 3% of samples lacking gender information (**Figure 1D**). Age distribution indicated that most Omicron samples were from the 19-40 years age group, followed by the 41-60 years age group, with the least number of samples from the under 1 year age group (**Figure 1E**). Epidemiological data against the fifth wave of COVID-19 in Pakistan was sourced from Johns Hopkins Coronavirus Resource Center (https://coronavirus.jhu.edu/region/pakistan) and visualized (**Figure 1F**), which corresponded to the data reporting trend (**Figure 1A**).

### 3.2 Genetic diversity and population differentiation

We conducted population genomics analysis to evaluate genetic diversity and population differentiation in the Omicron variant, specifically within Pakistani samples. Genetic diversity was quantified using theta-W and nucleotide diversity (Pi), revealing a low level of within-population genetic diversity (Pi = 0.00177 ± 0.00048; θW = 0.00270 ± 0.00091). The values of Pi and theta-W were not significantly different, indicating neutrality (Tajima, 1989), which was corroborated by the neutrality test (Tajima’s D = -1.27149, p > 0.10).

**Figure 2:**
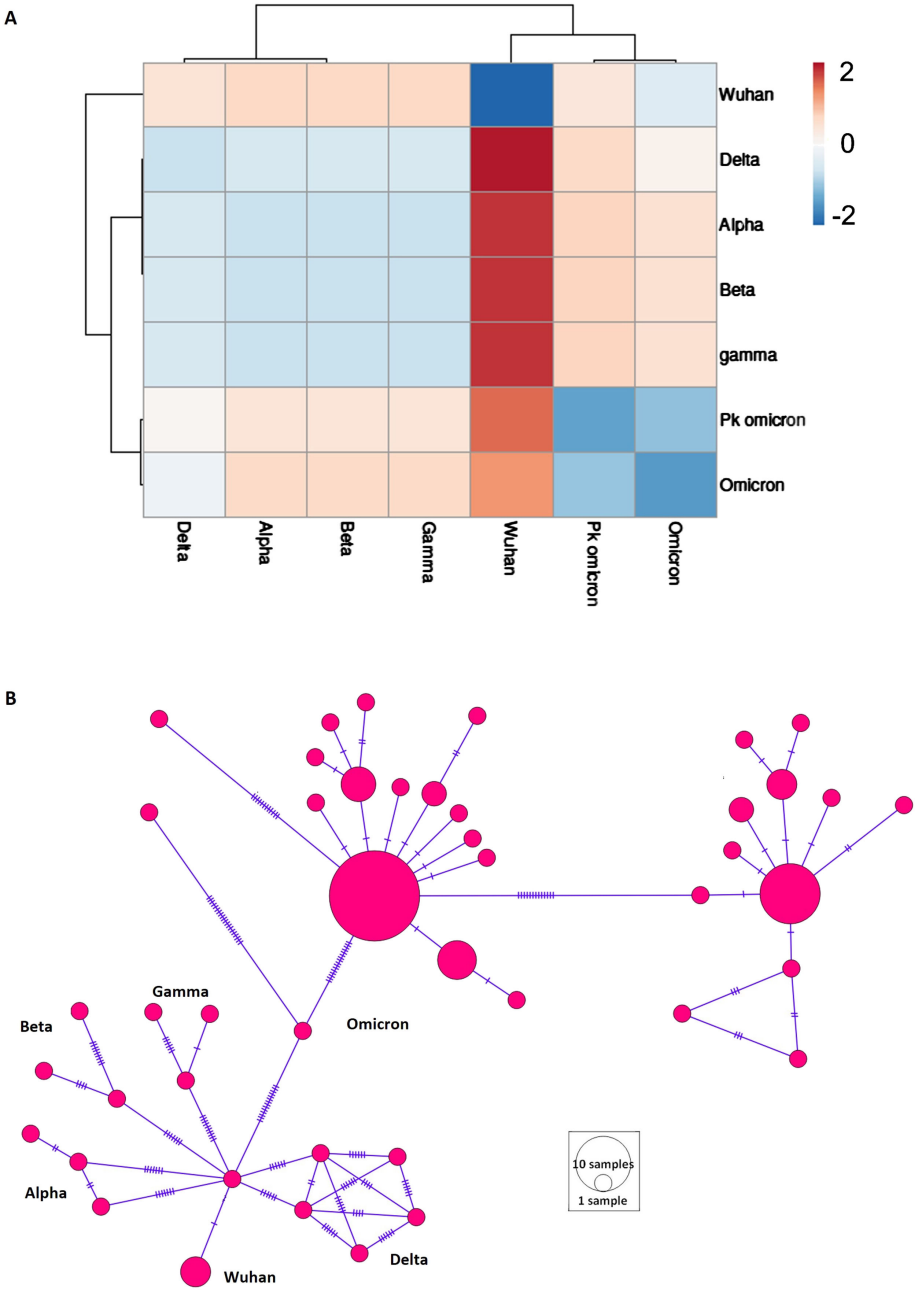
Population structure of different variant of concern (VOCs) of SARS-CoV-2. **(A)** Heatmap of population differentiation. Pakistani samples of Omicron (Pk omicron) were compared to different VOCs of SARS-CoV-2. **(B)** Network profiling of VOCs of SARS-CoV-2. Emergence of various VOCs from Wuhan1 variant can be seen.

Pairwise Fst values were computed to assess population structure across different populations (**Figure 2A**). The analysis showed that the Omicron variant shared similarities with the Delta variant and exhibited the most diversity from the Wuhan variant (**Figure 2A**). The Alpha, Beta, and Gamma variants showed minimal differentiation among themselves. A median-joining network was reconstructed to visualize haplotype distribution and recapitulate the evolutionary history of SARS-CoV-2 (**Figure 2B**). The network effectively depicted the population structure and traced the evolutionary history from the Wuhan origin to various VOCs, culminating in Omicron.

### 3.3 Structural features of the S protein of SARS-CoV-2 in Pakistani samples of omicron variant

**Figure 3:**
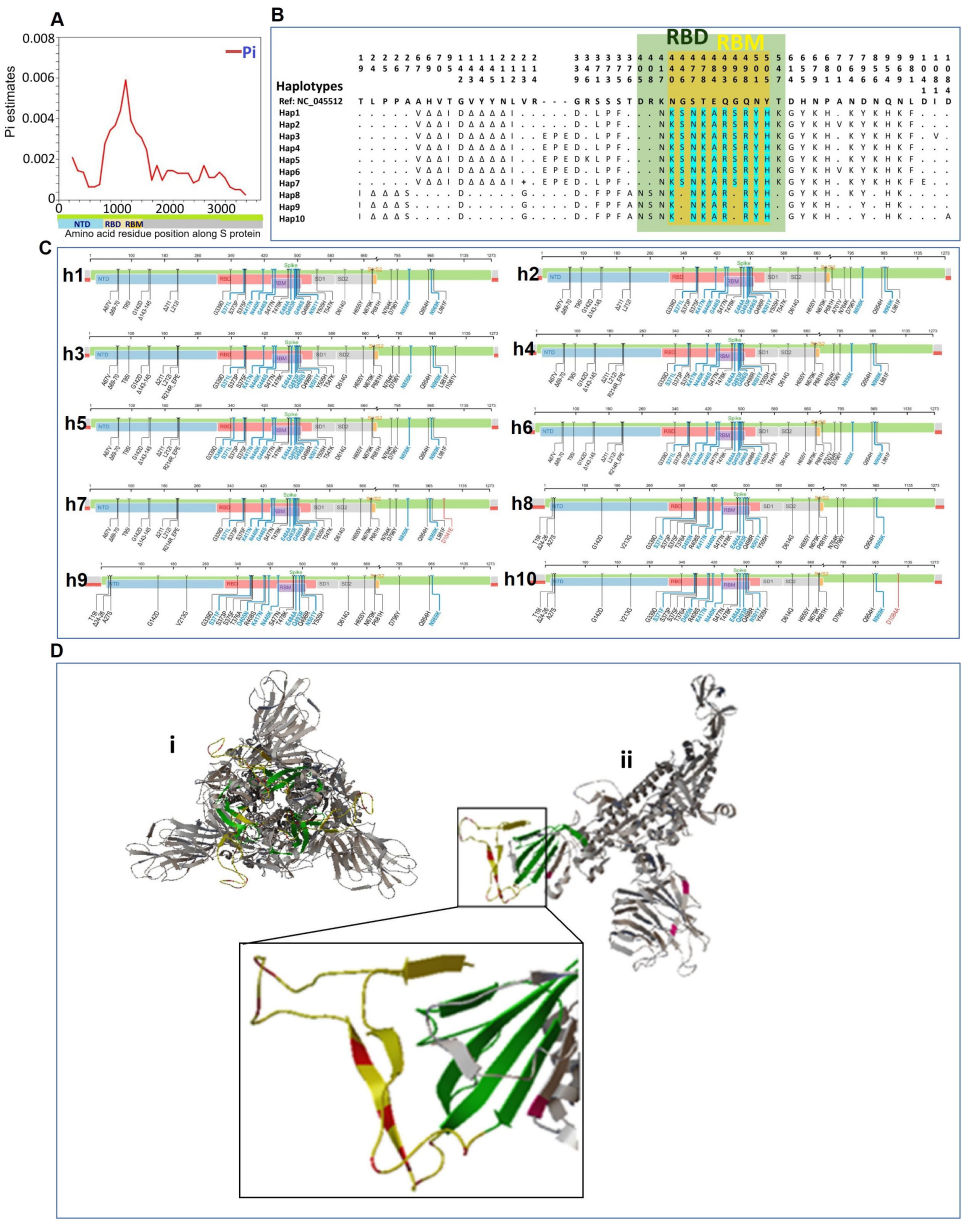
Structural features of the S protein of SARS-CoV-2 in Pakistani samples of Omicron variant. **(A)** Mapping of genetic diversity (Pi) along the S protein. **(B)** Variations among the 10 haplotypes of spike protein of Omicron in Pakistan. Dots (.) represent the amino acids residue similar to the reference sequence (GenBank accession number NC_045512). The color annotation: green, RBD (receptor binding domain); yellow, RBM (receptor binding motif)-region within the RBD; turquoise, mutations in RBM region. **(C)** Mapping of mutations for different haplotypes of Pakistani samples of Omicron. The complete sets of mutations for each of the ten haplotypes (h1-h10) are shown along the spike protein region. **(D)** Spike protein 3-D Structure and mutations of Pakistani Omicron samples. (i) The trimeric structure of full-length spike protein of Omicron. (ii) Monomer of spike protein with highlighted mutations. The region with highlighted mutations is shown in enlarged. Color codes: green color indicates the RBD; yellow color indicates RBM within the RBD; and red color shows the mutations in the RBM.

To analyze the S-protein structural features of Pakistani Omicron samples, we investigated the spectrum of variations in the S-protein region. The spike protein sequences from Pakistani Omicron samples ranged in length from 3804 to 3813 nucleotides (1267-1270 amino acids). We identified 41 polymorphic sites, comprising 16 parsimony informative sites and 25 singleton variable sites, along with 36 InDel sites. We also calculated and mapped the genetic diversity index Pi in 500-base windows along the S protein, revealing similar distribution of mutations across the S protein, except in the RBD region, which exhibited the highest fluctuation in Pi values (**Figure 3A**).

We examined the haplotype distribution of the spike protein in Pakistani Omicron samples (**Figure 3B**). MUSCLE-based MSA of amino acid sequences identified 53 amino acid mutations in S protein. Omicron-specific mutations were observed, including those in the RBD and RBM. These mutations were mapped along the S protein region for each of the haplotypes (**Figure 3C**). To visualize the mutations in the spike protein, 3-D structural models were generated (**Figure 3D**). The trimeric spike protein structure was simplified by deleting two chains, highlighting the respective mutations on a monomer.

### 3.4 Phylogenetic reconstruction

We first refined the dataset to achieve a more resolved, robust, and precise tree topology, avoiding a bulky and poorly resolved phylogeny. To this end, we initially employed a maximum-likelihood algorithm and optimized the phylogenetic reconstructions for an optimal tree topology. For a more detailed analysis under the Bayesian framework, the dataset was further refined by selecting a subset of sequences, removing those that were highly similar and those not relevant to the introduction of Omicron in Pakistan. This refinement reduced the dataset size, thereby conserving the extensive computational time and resources required for Bayesian simulations.

#### 3.4.1 Phylogenetic analysis of Omicron data from Pakistan

**Figure 4:**
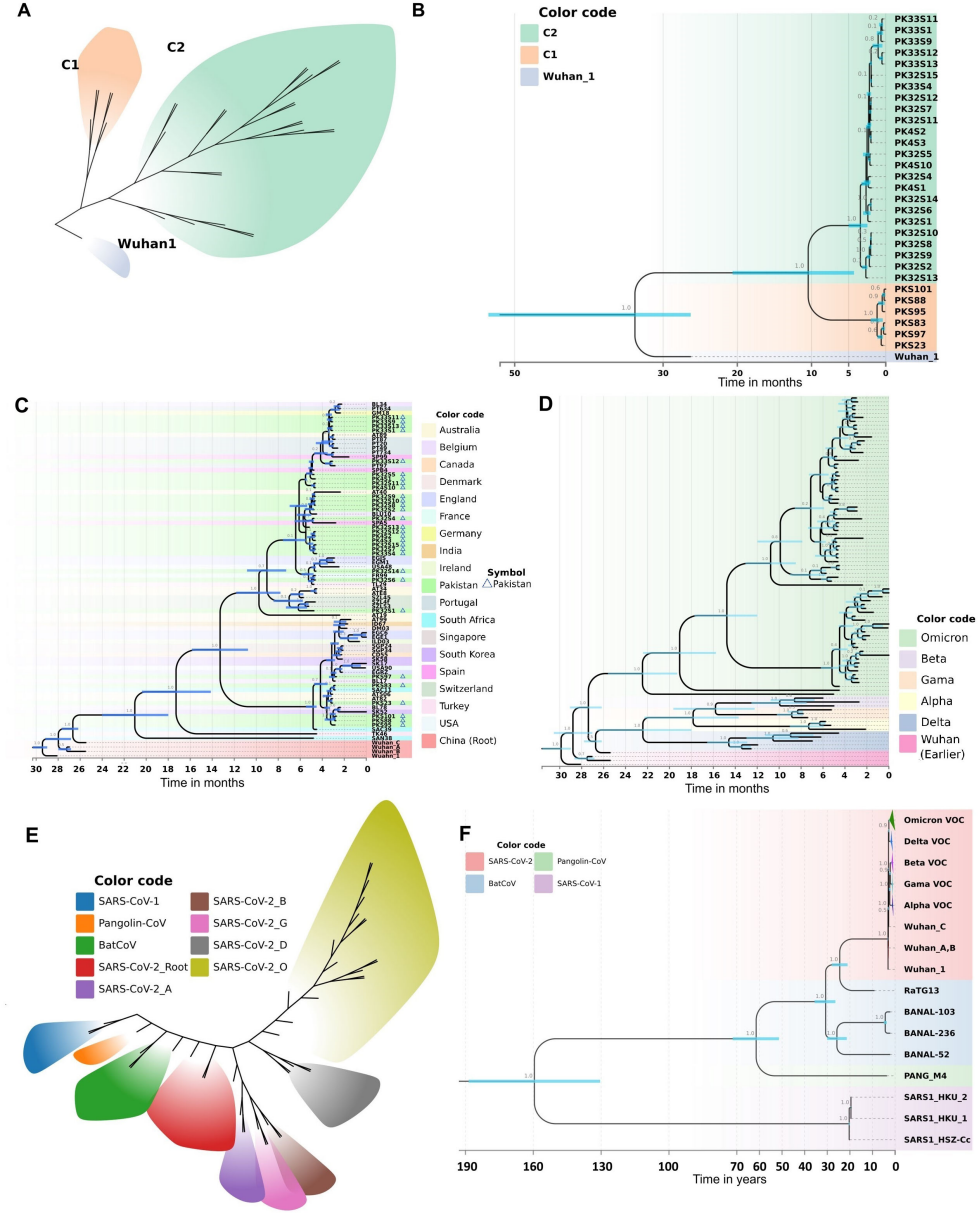
Molecular phylogeny and evolutionary history as revealed by a Bayesian framework during current study. **(A)** Clustering patterns of Pakistani samples of Omicron. **(B)** BEAST-MCMC chronogram showing the phylogenetic relationships and divergence time among the Omicron samples of Pakistan. Pakistani samples are represented by PK code. The scale bar indicates the time in months before February 15, 2022. C2: BA.1; C1: BA.2. **(C)** Evolutionary history of Pakistani Omicron samples as revealed by a time calibrated molecular phylogeny. The BEAST-MCMC chronogram showing the phylogenetic relationships and divergence time among the Omicron samples of Pakistan and other countries. The scale bar indicates the time in months before April 22, 2022. **(D)** Evolutionary history of Omicron VOC with other VOCs (Alpha, Beta, Gamma, Delta). The BEAST-MCMC chronogram showing the phylogenetic relationships and divergence time among the Omicron VOC with other VOCs samples. The scale bar indicates the time in months before April 22, 2022. **(E, F)** Putting all data into a greater context. Evolutionary history of SARS-CoV-2 with other coronaviruses (SARS-CoV-1, PANG21, BNL47, BNL45, BN44, RaTG13) revealed by a time calibrated molecular phylogeny reconstructed in Bayesian framework during current study. **(E)** Evolutionary divergence and clustering patterns of the data set. **(F)** The BEAST-MCMC chronogram showing the phylogenetic relationships and divergence time among the SARS-CoV-2 with other coronaviruses. The scale bar indicates the time in years before April 2022. Branch values show the Bayesian posterior probabilities. Node bars in blue color indicate the 95% HPD of node height.

To visualize the clustering patterns of Pakistani Omicron samples, we implemented a Bayesian framework to estimate a time-calibrated molecular phylogeny using MCMC algorithms. The best-fit evolutionary model for this dataset was the K3Pu+F model (**Supplementary Table 3**), as suggested by the ModelFinder tool. The phylogenetic tree revealed two major clades, C1 and C2 (**Figure 4A**). The time-resolved phylogeny illuminated the phylogenetic relationships and divergence of these clades (**Figure 4B**). Samples under Clade C1 were mainly from the BA.2 lineage, while those under Clade C2 were mainly from the BA.1 lineage. Clade C1, subdivided into two sub-clades, shared their MRCA in January 2022. Clade C2, larger and further divided into various sub-clades, shared their MRCA in December 2021. These findings suggest that Omicron was introduced into Pakistan from two different lineages, with Clade C2 spreading into multiple cities. The estimated clock rate for this dataset was 2.562E-3 mutations/site/year (95% HPD interval: 8.8067E-4 to 4.1462E-3).

#### 3.4.2 The evolutionary history of the Omicron variant in Pakistan

To elucidate the evolutionary history of the Omicron variant in Pakistan, we analyzed Pakistani Omicron samples alongside global Omicron data. This analysis involved reconstructing a time-calibrated phylogeny with the MCMC algorithms in a Bayesian framework. The resultant phylogenetic tree (**Figure 4C**) revealed five major clades. Clade 1, consisting of Wuhan samples, formed the base of the tree. The remaining four clades contained Omicron samples, with the earliest South African lineage at the base (**Figure 4C**). The time to the most recent common ancestor (TMRCA) for all Omicron clades was estimated to be around July 2020.

Clade C1 of Pakistani Omicron samples shared a TMRCA of December 2021, originating from a South African lineage isolated in late December 2021 (**Figure 4C**). Clade C2 samples were distributed across various sub-clades, with TMRCAs ranging from December 2021 to January 2022. Our analysis revealed that this clade was introduced into Pakistan via lineages from Thailand (December 2021), Spain (late December 2021), and Belgium (early January 2022).

We also observed the spread of Omicron from Pakistan to other countries. Clade C1 Pakistani samples contributed to the introduction of Omicron into South Korea and Belgium. Specifically, samples reported in April 2022 from South Korea and in late February 2022 from Belgium shared their MRCA with Pakistani samples from Clade C1 (**Figure 4C**). Similarly, Omicron from Clade C2 Pakistani samples was introduced to Switzerland and France (early January 2022), and to the USA, Australia, and England (late February 2022).

The estimated evolutionary rate for this dataset was 2.887E-3 mutations/site/year (95% HPD interval: 2.2543E-3 to 3.5517E-3). The best-fit evolutionary model for this dataset was the HKY+F+I model (**Supplementary Table 3**), as suggested by the ModelFinder tool. The divergence dates calculated in our phylogeny correspond to the recorded history of SARS-CoV-2, thus validating the robustness of our analysis.

To get deeper insights into the evolutionary history of Omicron VOC, we analyzed global Omicron data alongside other VOCs (Alpha, Beta, Gamma, Delta). The time frame for data collection was till April 22, 2022. A time-calibrated phylogeny revealed nine major clades (**Figure 4D**). Omicron formed a distinct clade, diverging from early Wuhan lineages in January 2020. Delta also formed a separate clade, diverging from Alpha, Beta, and Gamma in May-June 2020. The phylogeny within Omicron samples was fully resolved, with samples clustering into four major clades. The estimated clock rate for this dataset was 2.355E-3 mutations/site/year (95% HPD interval: 1.9124E-3 to 2.8422E-3). The best-fit evolutionary model for this dataset was the K3Pu+F+I model (**Supplementary Table 3**), as suggested by the ModelFinder tool. The divergence dates calculated in our phylogeny correspond to the recorded history of SARS-CoV-2, thus validating the robustness of our analysis.

To analyze the Pakistani samples in the context of the overall evolutionary history of SARS-CoV-2, we included data from other coronaviruses (SARS-CoV-1, PANG21, BNL47, BNL45, BN44, RaTG13). To visualize the evolutionary clustering patterns, we employed a Bayesian framework to estimate a time-calibrated molecular phylogeny using MCMC algorithms. The best-fit evolutionary model for this dataset was the TIM2+F+G4 model (**Supplementary Table 3**), as suggested by the ModelFinder tool. The sequences diverged into nine different clusters, with each of the SARS-CoV-2 VOCs differentiating into a distinct cluster (**Figure 4E**). The BEAST-MCMC chronogram displayed the phylogenetic relationships and divergence times among SARS-CoV-2 and other coronaviruses (**Figure 4F**). SARS-CoV-1 formed a distinct clade, sharing its MRCA with other coronaviruses approximately 154 years ago. RaTG13, closely related to Wuhan 1, shared an MRCA about 23 years ago. The estimated clock rate for this dataset was 2.378E-3 mutations/site/year (95% HPD interval: 1.9614E-3 to 2.8083E-3). The divergence dates calculated in our phylogeny correspond with the recorded history of SARS-CoV-2 and literature, thereby validating the robustness of our analysis.

### 3.5 Population dynamics of SARS-CoV-2: recapitulating the Omicron outbreak

**Figure 5:**
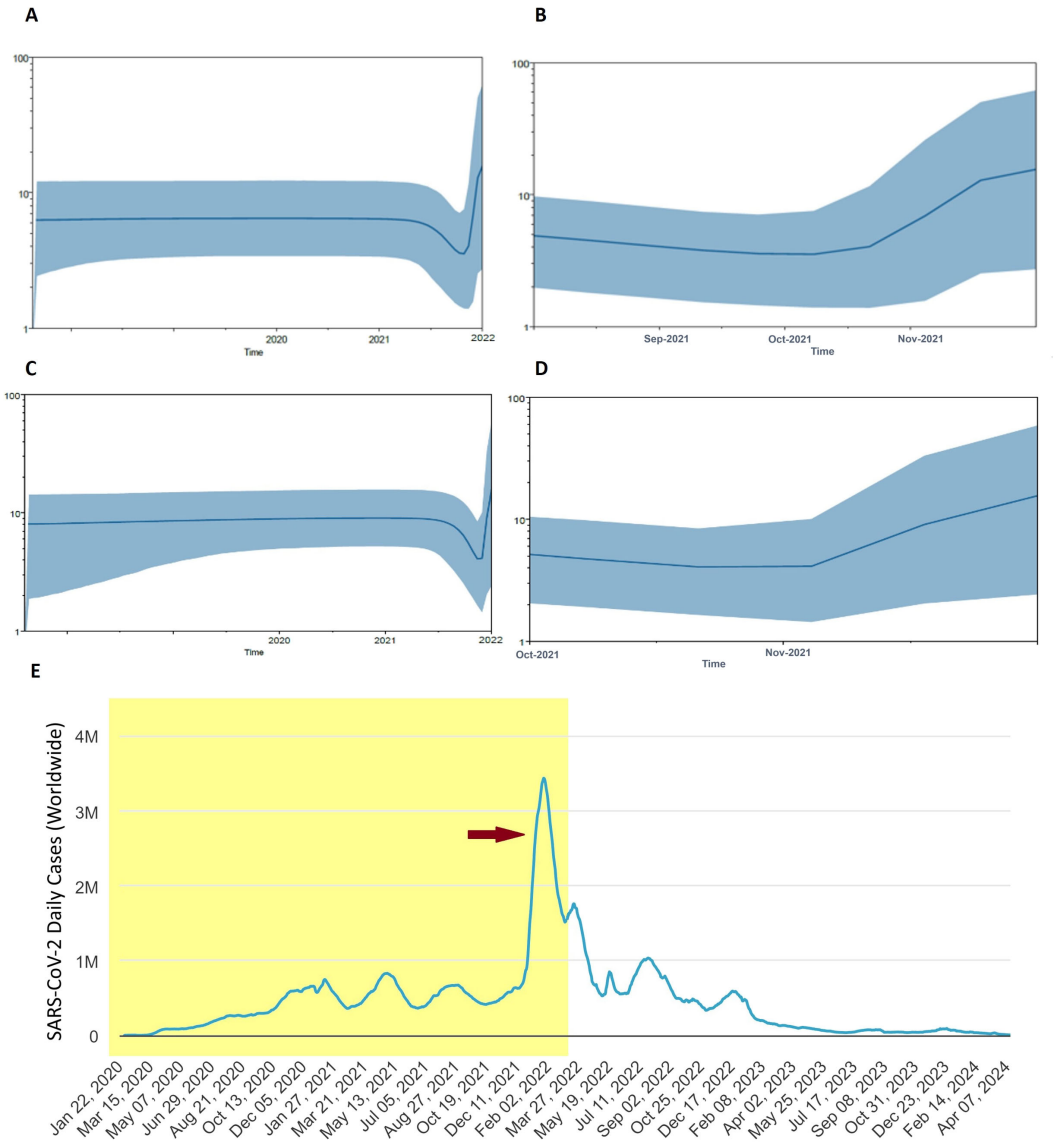
Population dynamics of Omicron pandemic. **(A)** Bayesian skyline plot (BSP) analysis for all Omicron data in the current study. **(B)** BSP analysis of Omicron from mid of 2021 to April 2022 is shown enlarged. **(C)** Overall BSP analysis of all SARS-CoV-2 in the current study. **(D)** BSP analysis of SARS-CoV-2 from August 2021 to April 2022 is shown enlarged. The blue line in the middle represents the mean estimate of the effective population size. Shaded region depicts the top and lower 95% confidence interval estimations. The y-axis shows the effective population size, while the x-axis shows time in years. **(E)** Worldwide Epidemiological data of COVID-19 (source: Worldometer, https://www.worldometers.info/coronavirus/). Red arrow indicates the Omicron outbreak. Shaded area corresponds to time span of BSP analysis.

To elucidate the past population dynamics of SARS-CoV-2, including the Omicron variant and other VOCs, we reconstructed a Bayesian skyline plot (BSP) (**Figure 5**). This analysis is crucial as it allows us to understand the temporal changes in the effective population size, providing insights into how the virus has spread and evolved over time. The BSP revealed a steady population growth rate of SARS-CoV-2 before 2021. However, this growth rate began to decline in mid-2021. A sudden expansion in population size was observed at the end of 2021, corresponding to the Omicron outbreak (**Figure 5A**). Further analysis focusing on the period from mid-2021 to April 2022 indicated a significant population expansion starting in November 2021 and continuing to increase, mirroring the rapid spread of the Omicron variant (**Figure 5B**). When analyzing the population dynamics of all SARS-CoV-2 data, including all major VOCs, similar trends were observed. There was a notable population expansion beginning in late 2021, consistent with the emergence and spread of Omicron (**Figures 5C, D**). This analysis successfully recapitulates the Omicron pandemic, demonstrating a clear temporal correlation between the observed population dynamics and the known epidemiological events (**Figure 5E**). The emergence of Omicron at the end of 2021 led to a sharp increase in the effective population size of the virus, highlighting the variant’s significant impact on the pandemic’s trajectory.

## 4 Discussion

In this study we elucidated genomic epidemiology and evolutionary dynamics of the Omicron variant of SARS-CoV-2 during the fifth wave of COVID-19 in Pakistan through comprehensive analysis of genetic diversity, population differentiation, and evolutionary history. The study provides crucial insights into how Omicron has evolved and spread within the Pakistani context, contributing to our understanding of its global impact.

The genomic analysis revealed a low level of genetic diversity within the population, as evidenced by the Pi value of 0.00177 ± 0.00048. This low genetic diversity suggests a recent introduction and rapid spread of the Omicron variant in Pakistan. The Omicron variant of SARS-CoV-2 harbors several mutations in its RBD and other regions, which significantly impact interactions with therapeutic antibodies and the immune system. All Omicron specific mutations (Zhao et al., 2022) were observed in our analysis including those found in RBD and RBM. These mutations result in the extremely high affinity of the Omicron RBD for the human ACE2 receptor, with notable changes including Q493R, T478K, S373P, N501Y, Q498R, S371L, and S375F (Chatterjee et al., 2023). The presence of naturally hydrophobic amino acids, such as leucine and phenylalanine, contributes to this enhanced affinity (Goutam Mukherjee et al., 2022). Some of these mutations facilitate the formation of salt bridges or hydrogen bonds, enhancing the binding of the spike protein to hACE2. However, mutations like K417N and E484A can significantly reduce polar contacts between Omicron and ACE2, counteracting some of the improved interactions created by other mutations (Cui et al., 2022; Han et al., 2022; Yin et al., 2022). A deeper examination of the crystal structures of the RBD–ACE2 complex of Omicron reveals a larger interaction surface area with the host compared to the Delta variant (Han et al., 2022). The RBD, directly involved in binding with hACE2, harbors ten significant mutations, thereby altering the affinity of the spike protein for the host receptor (Kumar et al., 2022). Among these mutations, T478K, Q498R, N440K, and Q493R are particularly important. These mutations, by replacing uncharged amino acid residues with positively charged lysine and arginine, enhance the stability of the RBD–hACE2 complex, thus improving the binding of the RBD to the human ACE2 receptor (Jung et al., 2022). In addition to these structural and binding implications, several Omicron mutations exhibit varying impacts on antibody susceptibility (Chen et al., 2022; Iketani et al., 2022; Kumar et al., 2023). G339D, a rare core mutation in the RBD, shows slight resistance to sotrovimab in laboratory assays. K417N, found in several VOCs including Beta, Gamma, and Omicron, significantly reduces ACE2 binding and confers resistance to etesivimab and casirivimab while retaining susceptibility to other monoclonal antibodies (mAbs). N440K, prevalent in Omicron, shows resistance to imdevimab and C135 mAbs targeting the RBD core but remains susceptible to sotrovimab and convalescent plasma. Conversely, S477N and T478K, common in Omicron, do not reduce susceptibility to FDA EUA-approved mAbs, whereas E484A significantly reduces susceptibility to several mAbs, including C121 and C144 (Jung et al., 2022). Overall, the mutations in the Omicron variant underscore its adaptive evolution and the ongoing challenges they pose for therapeutic and vaccine development efforts. Computational studies suggest that the RBD–hACE2 complex of Omicron is highly stable due to these mutations, contributing to its high transmissibility and significant impact on the global response to the pandemic (Jung et al., 2022).

The evolutionary history of the Omicron variant in Pakistan was elucidated through a time-calibrated phylogenetic analysis and Bayesian skyline plot. Our results indicate that Omicron was introduced into Pakistan via two distinct lineages, clades C1 (BA.2) and C2 (BA.1), which subsequently spread across the country. Specifically, C1 shared its most recent common ancestor (MRCA) in January 2022, originating from a South African lineage isolated in late December 2021. In contrast, clade C2 shared its MRCA in December 2021 and was introduced through lineages originating from Thailand, Spain, and Belgium. These findings suggest that the Omicron variant in Pakistan has multiple origins and entered the country through various sources. The observed spread of Omicron from Pakistan to other countries and vice versa underscores the necessity for global cooperation in controlling viral dissemination and monitoring emerging variants. The evolutionary history of Omicron, analyzed alongside other SARS-CoV-2 VOCs, revealed complex phylogenetic relationships and divergence patterns. The population differentiation analysis revealed that Omicron exhibited similarities with the Delta variant, indicating a potential evolutionary linkage. However, it displayed the most diversity compared to the Wuhan variant. Phylogenetic analysis highlighted that Omicron is the most divergent among all VOCs, forming a distinct monophyletic group with an extremely long branch length, sharing its MRCA with other VOCs early in 2020. This suggests that Omicron emerged from a lineage separate from other VOCs, possibly due to its accumulation of numerous mutations. Several hypotheses have been proposed regarding the origin of Omicron (Markov et al., 2023). One hypothesis posits that Omicron may have “cryptically propagated” in a population with inadequate viral monitoring and sequencing (Du et al., 2022). Another suggests that Omicron could have developed in a COVID-19 patient with a long-term infection, such as an immunocompromised individual, providing a favorable environment for intra-host viral adaptation (Callaway, 2021). A third hypothesis is that Omicron might have accumulated mutations in a nonhuman host before zoonotic transmission to humans (Shan et al., 2021).

The population dynamics analysis of SARS-CoV-2, as revealed by the BSP, offers a comprehensive view of the virus’s evolutionary trajectory. The analysis successfully recapitulated the pandemic trajectory, showing a steady growth rate before 2021, followed by a decline in mid-2021 and a subsequent sharp population expansion at the end of 2021 coinciding with the Omicron surge. The BSP showed a steady population growth rate before 2021, which is consistent with the initial rapid spread of COVID-19 following its emergence in late 2019. This rapid spread was a result of the virus’s high transmissibility and the lack of pre-existing immunity in the global population. As mass vaccination campaigns were rolled out worldwide in early 2021, a decline in the population growth rate of SARS-CoV-2 was observed starting mid-2021 (Paetzold et al., 2022; Watson et al., 2022). This decline aligns with the historical record of the pandemic, where widespread vaccination efforts significantly reduced transmission rates and curbed the spread of the virus. However, towards the end of 2021, the BSP revealed a sudden expansion in the population size of SARS-CoV-2, which corresponds to the emergence and spread of the Omicron variant (Karim and Karim, 2021; Tegally et al., 2022; Viana et al., 2022). This period marked a significant increase in cases globally, despite high levels of vaccination, due to Omicron’s enhanced transmissibility and ability to partially evade immunity conferred by previous infections and vaccinations. These results successfully recapitulate the Omicron pandemic, demonstrating a clear temporal correlation between the observed population dynamics and the known epidemiological events, thus underscore the importance of continuous genomic surveillance and population dynamics studies. Such analyses are crucial for understanding how new variants emerge and spread, providing valuable insights that can inform public health strategies and interventions.

While our study provides comprehensive insights into the genomic epidemiology and evolutionary dynamics of the Omicron variant in Pakistan, it is essential to acknowledge certain limitations, such as the potential sampling bias due to unequal geographic distribution and temporal coverage of the retrieved samples, which may not fully capture the genetic diversity of the virus across the entire population. Moreover, the reliance on publicly available sequences from databases may introduce biases due to underreporting or variability in data quality and completeness. Recognizing these limitations helps provide a balanced view of our findings and underscores the need for continuous and comprehensive genomic surveillance efforts to inform effective public health responses.

In conclusion, this study presents a comprehensive examination of the genetic diversity, genomic epidemiology, and evolutionary dynamics of the Omicron variant in Pakistan during the fifth wave of COVID-19. Our findings underscore the complex evolutionary landscape of Omicron within the Pakistani population, highlighting the introduction of two distinct lineages—clades C1 (BA.2) and C2 (BA.1)—into the country. Specifically, clade C1 originated from a South African lineage in January 2022, while clade C2 emerged in December 2021, introduced via lineages from Thailand, Spain, and Belgium. These results illustrate the multiple origins of Omicron in Pakistan and its subsequent spread, both domestically and internationally. Notably, this study offers valuable insights into the transnational transmission patterns and adaptive evolution of Omicron, with important implications for genomic surveillance and public health strategies in Pakistan.

## Data Availability

The original contributions presented in the study are included in the article/**Supplementary Material**. Further inquiries can be directed to the corresponding author/s.
