# Supplementary material for: Genomic epidemiology and evolutionary dynamics of the Omicron variant of SARS-CoV-2 during the fifth wave of COVID-19 in Pakistan
Source: Front Cell Infect Microbiol. 2024 Oct 22;14:1484637. doi: 10.3389/fcimb.2024.1484637 (PMC11534695; doi:10.3389/fcimb.2024.1484637)
Supplement: Supplementary file 2 [file Table2.docx]

**Supplementary Table 2.** Details of samples shown in the figures

| **Accession No.** | **Location** | **Date** | **Code** |
| --- | --- | --- | --- |
| NC_045512 | China, Hubei | 12/16/2019 | Wuhan_1 |
| MT_259226 | China | 1/10/2020 | Wuhan A |
| MT_304483 | China | 3/1/2020 | Wuhan B |
| MT_276323 | China | 2/28/2020 | Wuhan C |
| AY_394995 | China | 11/16/2002 | SARS1 |
| GU_553364 | China | 6/7/2003 | SARSA |
| GU_553363 | China | 6/7/2003 | SARSB |
| EPI_ISL_410721 | China | 3/2019 | PANG21 |
| EPI_ISL_4302644 | Laos | 7/5/2020 | BNL44 |
| EPI_ISL_4302645 | Laos | 7/7/2020 | BNL45 |
| EPI_ISL_4302647 | Laos | 7/10/2020 | BNL47 |
| EPI_ISL_13350790 | Brazil | 8/16/2021 | GAM08 |
| EPI_ISL_9274958 | Brazil | 12/10/2021 | GAM23 |
| EPI_ISL_13228901 | Brazil | 9/4/2021 | GAM62 |
| EPI_ISL_13159113 | India | 12/29/2021 | DEL14 |
| EPI_ISL_13390364 | India | 11/16/2021 | DEL93 |
| OP430920 | India | 5/06/2021 | DEL44 |
| OP647409 | Vietnam | 9/29/2021 | DEL63 |
| EPI_ISL_6699711 | South Africa | 10/20/2021 | BET01 |
| EPI_ISL_7605583 | South Africa | 10/15/2021 | BET72 |
| EPI_ISL_5134427 | UK | 10/8/2021 | ALPHA |
| EPI_ISL_10364895 | UK | 1/31/2022 | ALPHA |
| EPI_ISL_6026865 | UK | 10/28/2022 | ALPHA |
| EPI_ISL_8336432 | Turkey | 12/31/2021 | TK46 |
| EPI_ISL_12396994 | Belgium | 2/22/2022 | BL78 |
| EPI_ISL_12681773 | South Korea | 4/22/2022 | SK52 |
| EPI_ISL_11262669 | Pakistan | 2/12/2022 | PKS88 |
| EPI_ISL_11262675 | Pakistan | 2/14/2022 | PKS101 |
| EPI_ISL_11055872 | Belgium | 2/10/2022 | BL17 |
| EPI_ISL_10664009 | South Korea | 1/28/2022 | SK58 |
| EPI_ISL_12317002 | Australia | 3/3/2022 | AT82 |
| EPI_ISL_12850812 | Denmark | 3/7/2022 | DM03 |
| EPI_ISL_9686877 | South Africa | 12/30/2021 | SAC39 |
| EPI_ISL_9596947 | Singapura | 1/30/2022 | SGP34 |
| EPI_ISL_9990850 | South Korea | 1/10/2022 | SAC11 |
| EPI_ISL_12137135 | Singapura | 3/3/2022 | SGP24 |
| EPI_ISL_12316985 | Australia | 3/3/2022 | AT506 |
| EPI_ISL_10783120 | Canada | 1/27/2022 | CD55 |
| EPI_ISL_11262662 | Pakistan | 2/11/2022 | PKS83 |
| EPI_ISL_11262674 | Pakistan | 2/14/2022 | PKS97 |
| EPI_ISL_11262698 | Pakistan | 2/3/2022 | PKS23 |
| EPI_ISL_12918296 | England | 3/28/2022 | EGRZ |
| EPI_ISL_8903317 | Ireland | 12/19/2021 | ILD03 |
| EPI_ISL_12769870 | Australia | 3/15/2022 | AT99 |
| EPI_ISL_13065660 | England | 4/20/2022 | EGE1 |
| EPI_ISL_13065644 | England | 4/20/2022 | EGC6 |
| EPI_ISL_11262726 | Pakistan | 2/11/2022 | PKS95 |
| EPI_ISL_13086514 | South Korea | 4/19/2022 | SK17 |
| EPI_ISL_12954599 | India | 3/9/2022 | ID67 |
| EPI_ISL_12927664 | USA | 12/31/2021 | USA90 |
| EPI_ISL_11055860 | Belgium | 1/26/2022 | BL34 |
| EPI_ISL_9083481 | Belgium | 12/30/2021 | BLU10 |
| EPI_ISL_9300755 | Portugal | 1/16/2022 | PT49 |
| EPI_ISL_11686009 | Australia | 1/6/2022 | AT89 |
| EPI_ISL_10122563 | Portugal | 2/7/2022 | PT734 |
| EPI_ISL_9606418 | Portugal | 1/23/2022 | PT634 |
| EPI_ISL_10122550 | Portugal | 2/7/2022 | PT20 |
| EPI_ISL_8240503 | Pakistan | 12/25/2021 | PK32S5 |
| EPI_ISL_8240505 | Pakistan | 12/27/2021 | PK32S7 |
| EPI_ISL_8240506 | Pakistan | 12/26/2021 | PK32S8 |
| EPI_ISL_8240507 | Pakistan | 12/26/2021 | PK32S9 |
| EPI_ISL_8240509 | Pakistan | 12/27/2021 | PK32S11 |
| EPI_ISL_8240510 | Pakistan | 12/27/2021 | PK32S12 |
| EPI_ISL_8240511 | Pakistan | 12/12/2021 | PK32S13 |
| EPI_ISL_8240513 | Pakistan | 12/28/2021 | PK32S15 |
| EPI_ISL_8650048 | Pakistan | 1/3/2022 | PK33S1 |
| EPI_ISL_8650049 | Pakistan | 12/30/2021 | PK33S4 |
| EPI_ISL_8650052 | Pakistan | 1/3/2022 | PK33S9 |
| EPI_ISL_8650053 | Pakistan | 1/3/2022 | PK33S11 |
| EPI_ISL_8650054 | Pakistan | 1/3/2022 | PK33S12 |
| EPI_ISL_8650055 | Pakistan | 1/3/2022 | PK33S13 |
| EPI_ISL_8767206 | Pakistan | 12/27/2021 | PK4S2 |
| EPI_ISL_8767207 | Pakistan | 12/27/2021 | PK4S3 |
| EPI_ISL_8767208 | Pakistan | 12/28/2021 | PK4S10 |
| EPI_ISL_8240502 | Pakistan | 12/25/2021 | PK32S4 |
| EPI_ISL_8240504 | Pakistan | 12/26/2021 | PK32S6 |
| EPI_ISL_8240512 | Pakistan | 12/28/2021 | PK32S14 |
| EPI_ISL_11686014 | Australia | 1/23/2022 | AT40 |
| EPI_ISL_9003407 | Portugal | 1/11/2022 | PT97 |
| EPI_ISL_8767205 | Pakistan | 12/27/2021 | PK4S1 |
| EPI_ISL_11225857 | France | 12/27/2021 | FR99 |
| EPI_ISL_8594403 | Thailand | 12/23/2021 | TL29 |
| EPI_ISL_9003415 | Portugal | 1/10/2022 | PT87 |
| EPI_ISL_13091612 | Spain | 3/12/2022 | SP99 |
| EPI_ISL_10931942 | Spain | 12/24/2021 | SPB4 |
| EPI_ISL_10931949 | Spain | 1/13/2022 | SPA5 |
| EPI_ISL_7566875 | Swaziland | 11/30/2022 | SZL45 |
| EPI_ISL_8240498 | Pakistan | 12/25/2021 | PK32S1 |
| EPI_ISL_8240500 | Pakistan | 12/25/2021 | PK32S2 |
| EPI_ISL_8240508 | Pakistan | 12/26/2021 | PK32S10 |
| EPI_ISL_13166683 | USA | 1/18/2022 | USA48 |
| EPI_ISL_10305576 | Australia | 12/31/2021 | AT34 |
| EPI_ISL_10263727 | Australia | 12/30/2021 | ATE8 |
| EPI_ISL_12999929 | England | 2/9/2022 | EGM1 |
| EPI_ISL_12999924 | England | 2/9/2022 | EGJS |
| EPI_ISL_8684425 | Swaziland | 12/26/2021 | SZL4f |
| EPI_ISL_8684429 | Swaziland | 12/10/2021 | SZL53 |
| EPI_ISL_10585293 | Australia | 2/20/2022 | AT19 |
| EPI_ISL_7337481 | South Africa | 11/22/2021 | SAN38 |
| EPI_ISL_10305576 | Australia | 12/31/2021 | AT34 |
| EPI_ISL_10263727 | Australia | 12/30/2021 | ATE8 |
| EPI_ISL_7337481 | South Africa | 11/22/2021 | SAN38 |
| EPI_ISL_8685512 | Germany | 2/22/2022 | GM18 |
| EPI_ISL_10585293 | Australia | 2/20/2022 | AT19 |
