# Supplementary material for: Genomic epidemiology and evolutionary dynamics of the Omicron variant of SARS-CoV-2 during the fifth wave of COVID-19 in Pakistan
Source: Front Cell Infect Microbiol. 2024 Oct 22;14:1484637. doi: 10.3389/fcimb.2024.1484637 (PMC11534695; doi:10.3389/fcimb.2024.1484637)
Supplement: Supplementary file 3 [file Table3.docx]

**Supplementary file 3**

**Results for evolutionary models**

1. K3Pu+F

| Rate parameter R of K3Pu+F | |
| --- | --- |
| A-C: | 1.0000 |
| A-G: | 1.6595 |
| A-T: | 0.1703 |
| C-G: | 0.1703 |
| C-T: | 1.6595 |
| G-T: | 1.0000 |

1. HKY+F+I

| Rate parameter R of HKY+F+I | |
| --- | --- |
| A-C: | 1.0000 |
| A-G: | 2.1364 |
| A-T: | 1.0000 |
| C-G: | 1.0000 |
| C-T: | 2.1364 |
| G-T: | 1.0000 |

1. K3Pu+F+I

| Rate parameter R of K3Pu+F+I | |
| --- | --- |
| A-C: | 1.0000 |
| A-G: | 1.6125 |
| A-T: | 0.4632 |
| C-G: | 0.4632 |
| C-T: | 1.6125 |
| G-T: | 1.0000 |

1. TIM2+F+G4

| Rate parameter R of TIM2+F+G4 | |
| --- | --- |
| A-C: | 1.6788 |
| A-G: | 3.5006 |
| A-T: | 1.6788 |
| C-G: | 1.0000 |
| C-T: | 6.8057 |
| G-T: | 1.0000 |
